# The Simulation of Mode Control for a Photonic Lantern Adaptive Amplifier

**DOI:** 10.3390/mi15111342

**Published:** 2024-10-31

**Authors:** Yuxuan Ze, Pengfei Liu, Hanwei Zhang, Yanyang Hu, Lianchuang Ding, Baozhu Yan, Jiangbin Zhang, Qiong Zhou, Wenguang Liu

**Affiliations:** 1 College of Advanced Interdisciplinary Studies, National University of Defense Technology, Changsha 410073, China; zeyuxuan18@nudt.edu.cn (Y.Z.); zhanghanwei100@163.com (H.Z.); ceastbons@163.com (Y.H.); dlc0192@163.com (L.D.); azhu711@163.com (B.Y.); zhangjiangbin1@163.com (J.Z.); qinqin0416@hotmail.com (Q.Z.); 2 Nanhu Laser Laboratory, National University of Defense Technology, Changsha 410073, China; 3 Hunan Provincial Key Laboratory of High Energy Laser Technology, National University of Defense Technology, Changsha 410073, China

**Keywords:** photonic lantern, mode control, fiber laser, adaptive optics

## Abstract

A photonic lantern is a low-loss device that connects a single multimode waveguide to multiple single-mode waveguides and can enhance the beam quality of a fiber laser by adaptively controlling the optical parameters (amplitude, phase, polarization) at the input. In this work, we combined the gains and losses of individual modes within the fiber amplifier and introduced a mode content parameter at the amplifier’s output as an evaluation function to simulate mode control effects. Mode competition within the gain fiber can degrade the control effect of the fundamental mode and lead to it taking a longer time for the control to converge. Optimal parameters, such as the gain fiber length and pumping method, were identified to improve control effectiveness. Specifically, an optimal gain fiber length of 8 m was determined, and backward pumping was found to achieve higher pumping efficiency and better control results. The system demonstrated significant power amplification potential and could stabilize mode control under different pumping powers ranging from 50 W to 5 kW. In conclusion, our research demonstrates that an adaptive fiber amplifier based on a photonic lantern can achieve a stable, high-power, large-mode-field, near-fundamental-mode output from the gain fiber. Although mode competition within the gain fiber can degrade the control effect of the fundamental mode and cause the control to take a longer time to converge, these aspects should be further studied to improve the control’s effectiveness. These findings contribute to the development of advanced simulation models that guide high-power mode control experiments and deepen our understanding of physical processes in science and technology.

## 1. Introduction

From the 1990s to around 2010, the output power of near-diffraction-limited fiber lasers underwent a substantial increase, but further power enhancement has been subject to serious constraints from nonlinear effects [1,2,3]. Traditionally, to elevate the threshold of nonlinear effects, the approach of expanding the mode field area is commonly employed [4,5]. However, with the increase in the mode field area, the number of modes that can exist within the fiber is also increasing, and as the power of the fiber laser is increased, the heat generated within the fiber will lead to a change in its refractive index, facilitating mode coupling at kilohertz rates, known as transverse mode instability (TMI) [6]. This phenomenon can severely degrade beam quality due to the coupling between the fundamental mode and higher-order modes within the gain fiber. Traditional methods to suppress higher-order modes, such as intensifying the bending loss and using partially doped fibers, have their limitations, including energy loss and reduced laser efficiency, respectively, ultimately lowering the thresholds of other nonlinear effects [7,8,9,10]. Thus, there is an urgent need for innovative modal control strategies to enhance the suppression of higher-order modes and increase the TMI threshold in large-mode-area fibers, which is where photonic lanterns emerge as a promising solution.

A photonic lantern is a low-loss device that connects multiple single-mode waveguides to a single multimode waveguide. Typically, it is fabricated by confining multiple single-mode fibers within a low-refractive-index glass tube and then fusing and tapering them under adiabatic conditions before they are fused to a specific multimode fiber [11,12,13,14]. Initially, the application of photonic lanterns was within the fields of astronomy and spectroscopy [15], but due to their unique mode evolution characteristics and excellent adaptability to optical systems, they have gradually found applications in fiber lasers. A major feature of a photonic lantern is that the input light parameter characteristics (amplitude, phase, and polarization) of each single-mode end determine the mode composition of the output end of the multimode fiber, and by changing these parameter characteristics at the input end, a mode-specific output can be theoretically achieved at the output end of the photonic lantern. Previous studies have demonstrated that by combining photonic lantern technology with large-mode-area fiber lasers and utilizing the input-to-output decision relationship of the photonic lantern, stable mode output in large-mode-area fibers can be achieved through controlling the optical parameters at the input of the photonic lantern [16].

Currently, simulations of mode control in photonic lanterns have primarily focused on mode control within photonic lantern devices themselves, without considering the impact of fiber amplifiers [17]. Experimentally, the mode control ability of photonic lanterns at a low power has been verified to be extremely strong, enabling a stable, near-fundamental-mode output even in 50-micron large-mode-area fibers [16]. The latest research also suggests that mode control can be achieved in kW-level photonic lantern amplifiers [18]. However, simulation analyses of high-power, large-mode-field photonic lantern lasers are scarce. In fiber amplifiers, the competition between different modes can lead to varying control effects. In this paper, we combine the gains and losses of individual modes within the fiber amplifier and introduce a mode content parameter at the amplifier’s output as an evaluation function to simulate the mode control effects of a photonic lantern amplifier. The simulation results demonstrate that the photonic lantern amplifier can effectively control the modes in large-mode-area fibers, producing kilowatt-level near-fundamental-mode beams. Furthermore, our simulation results suggest that an adaptive control system based on photonic lantern amplifiers holds significant potential for precise mode control and beam quality optimization, which is particularly suitable for fiber lasers with higher powers and larger mode areas.

## 2. Materials and Methods

**Figure 1:**
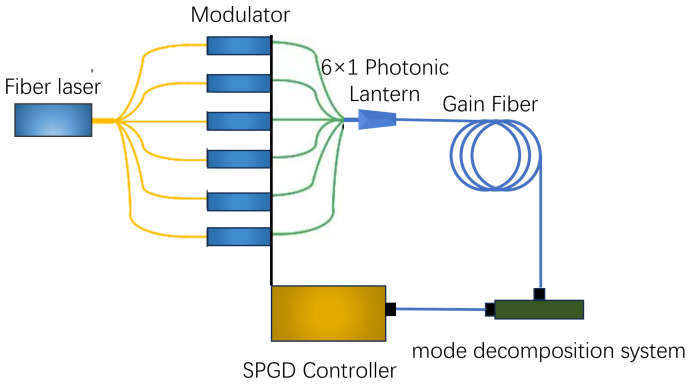
An adaptive fiber amplifier based on a photonic lantern.

An adaptive fiber amplifier based on a photonic lantern is illustrated in Figure 1. The broadened laser beam enters a single-mode fiber (SMF), and a beam splitter divides it into several separate beams. These beams then enter a lithium niobate phase modulator. When light is introduced at the input end of a photonic lantern, it experiences low-loss and high-efficiency mode coupling, resulting in a novel mode superposition field at the output end, referred to as mode evolution. The photonic lantern functions as a modulator for the seed light within the adaptive optics system. Light from the pigtail of the photonic lantern is directed into the amplifier for amplification, where parameters such as amplitude, phase, and polarization are meticulously adjusted to modify the modal components of the seed light, thereby enabling precise control over the output of the amplifier. The laser output from the photonic lantern enters a segment of the gain fiber which can amplify the signal light through either forward or backward pumping. At the output end of the gain fiber, feedback is introduced as an evaluation function for the Stochastic Parallel Gradient Descent (SPGD) control, which can be chosen from parameters such as the power in the bucket, the beam quality m^2^ factor, or the mode content.

The theory of photonic lantern mode control and the SPGD algorithm have been extensively reported; for more details, please refer to the relevant literature [17,19]. Our work differs from previous studies in that we consider the mode competition within the gain fiber. The power distribution and output of different modes within the fiber laser can be obtained by simply solving the rate equation, the transport equation, and the boundary equation. In this simulation work, the mode coupling effect is not considered. As we know, the coupling between different modes is mainly due to the nonlinear effects caused by thermal effect. In this simulation work, the diameter of the active fiber is 50 μm, and the largest pumping power is 5000 W; therefore, the heat flux density is still low enough that the thermal effect is not significant, as it is comparative to the thermal effect of an 800 W pumping power and a 20 μm core diameter. Thus, in this study, we have not accounted for mode coupling effects. In the following work, we will continue to improve our model, for example, by considering the nonlinear effects caused by thermal effects, TMI, etc., to study the mode control effects of photonic lantern amplifiers at higher powers.

In this model, the rate equation can be expressed as

(1) $$\begin{array}{c} \frac{dN_{1}\left(z\right)}{dt}=\frac{\Gamma_{p}\lambda_{p}\left[P_{p}^{+}\left(z\right)+P_{p}^{-}\left(z\right)\right]}{hcA_{c}}\left(\sigma_{ap}+\sigma_{as}\right)\left[N-N_{1}\left(z\right)\right]\\ -\frac{\sum_{i}\Gamma_{si}\lambda_{s}\left[P_{si}^{+}\left(z\right)+P_{si}^{-}\left(z\right)\right]}{hcA_{c}}\left(\sigma_{ep}+\sigma_{es}\right)N_{1}\left(z\right)-\frac{N_{1}\left(z\right)}{\tau }, \end{array}$$

where $N_{1}$ is the population density of the upper energy level of Yb ions. τ is the spontaneous lifetime of the energy level. *h* is Planck’s constant and *c* is the speed of light. *N* represents the doping concentration of Yb ions. Ac denotes the core area of the fiber. $P_{p}^{+}$ and $P_{p}^{-}$ are the pump powers in the forward and backward directions, respectively. $P_{si}^{+}$ and $P_{si}^{-}$ are the signal powers of the $i^{th}$ transverse mode in the forward and backward directions, respectively. $A_{c}$ denotes the core area of the fiber. $\Gamma_{p}$ and $\Gamma_{si}$ are the overlapping factors of the pump and the laser signal of the $i^{th}$ mode, respectively. $\sigma_{ap}$ ($\sigma_{ep}$) and $\sigma_{as}$ ($\sigma_{es}$) are the pump absorption (emission) and signal absorption (emission) cross-sections, respectively.

**Figure 2:**
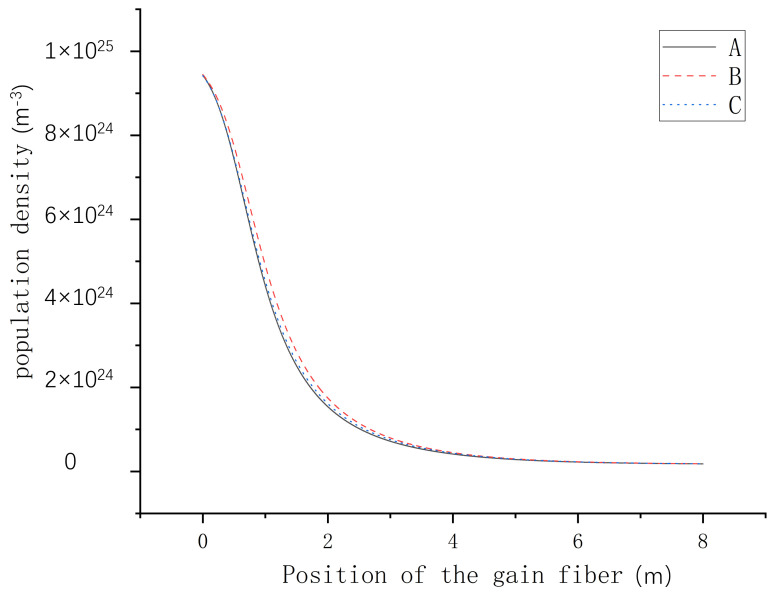
Distribution of population density with different mode contents. Line A: the seed light consists of the $LP_{01}$ mode. Line B: the seed light consists of the $LP_{11}$ mode. Line C: the seed light is composed of the $LP_{01}$ mode and the $LP_{11}$ mode in a 1:1 ratio.

In our previous simulation, we found that the modal content of the seed light had no significant impact on the amplifier’s population density, so we can consider the population density to be time-independent. In order to further prove the validity of the theory, we also tested the changes in population density after injecting the gain fiber with different mode contents, and the results are shown in Figure 2. Line A illustrates the variation in population density when the fundamental mode serves as the control target, Line B depicts the change in population density with the $LP_{11}$ mode is the control target, and Line C represents the alteration in population density when both the fundamental mode and $LP_{11}$ mode are simultaneously injected into the fiber at a 1:1 ratio. The comparison shows that there is no significant change in population density after different modes enter the gain fiber, which proves the effectiveness of the theoretical model of the amplification system.

For a continuous-wave fiber laser, $\frac{dN_{1}\left(z\right)}{dt}=0$. Then, $N_{1}\left(z\right)$ can be attained.

In this model, the propagation equation can be written as

(2) $$\begin{array}{c} \pm \frac{dP_{p}^{\pm }\left(z\right)}{dz}=-\Gamma_{p}\left[\sigma_{ap}\left(z\right)\left(N-N_{1}\left(z\right)\right)-\sigma_{ep}N_{1}\left(z\right)\right]P_{p}^{\pm }\left(z\right)-\alpha_{p}P_{p}^{\pm }\left(z\right)\\ \pm \frac{dP_{si}^{\pm }\left(z\right)}{dz}=\Gamma_{si}\left[\sigma_{es}N_{1}\left(z\right)-\sigma_{as}\left(N-N_{1}\left(z\right)\right)\right]P_{si}^{\pm }\left(z\right)-\alpha_{s}P_{si}^{\pm }\left(z\right)+\Gamma_{si}\sigma_{es}N_{1}\left(z\right)\frac{2hc^{2}}{\lambda_{s}^{3}}\Delta \lambda_{e} \end{array}$$

where $\alpha_{p}$ and $\alpha_{si}$ are the propagation losses of the active fiber at the pump wavelength and each mode’s laser wavelength (including the intrinsic absorption of the fiber and bending loss), respectively. $\Delta \lambda_{e}$ is the spontaneous emission bandwidth of YDF.

The gain coefficient of mode *i* can be written as

(3) $$g_{i}\left(z\right)=\Gamma_{si}\left[\sigma_{es}N_{1}\left(z\right)-\sigma_{as}\left(N-N_{1}\left(z\right)\right)\right]P_{si}^{\pm }\left(z\right)-\alpha_{s}P_{si}^{\pm }\left(z\right)+\Gamma_{si}\sigma_{es}N_{1}\left(z\right)\frac{2hc^{2}}{\lambda_{s}^{3}}\Delta \lambda_{e}$$

The power of mode *i* at point *l* in the active fiber can be written as

(4) $$P_{si}=\int_{0}^{l}P_{i0}\;g_{i}\left(z\right)dz$$

where $P_{i0}$ is the power of mode *i* at the input of the active fiber. Thus, the output beam profile from the gain fiber at point *l* is a coherent superposition of each of the supported modes, which can be described as

(5) $$\psi \left(x,y\right)=\sum_{i}\sqrt{P_{si}}\psi_{i}\left(x,y\right)exp\left(-i\varphi_{i0}\right)exp\left(-\left(\beta_{i}-\beta_{0}\right)l\right)$$

where $P_{si}$ is the power of mode *i*; $\psi_{i}$ is the beam profile of mode *i*; $\varphi_{i0}$ is the phase of mode *i* at the input of the gain fiber, which is determined by phase modulator; $\beta_{0}$ is the propagation constant of the fundamental mode; $\beta_{i}$ is the propagation constant of the high-order mode; and *l* is the point in the gain fiber.

**Figure 3:**
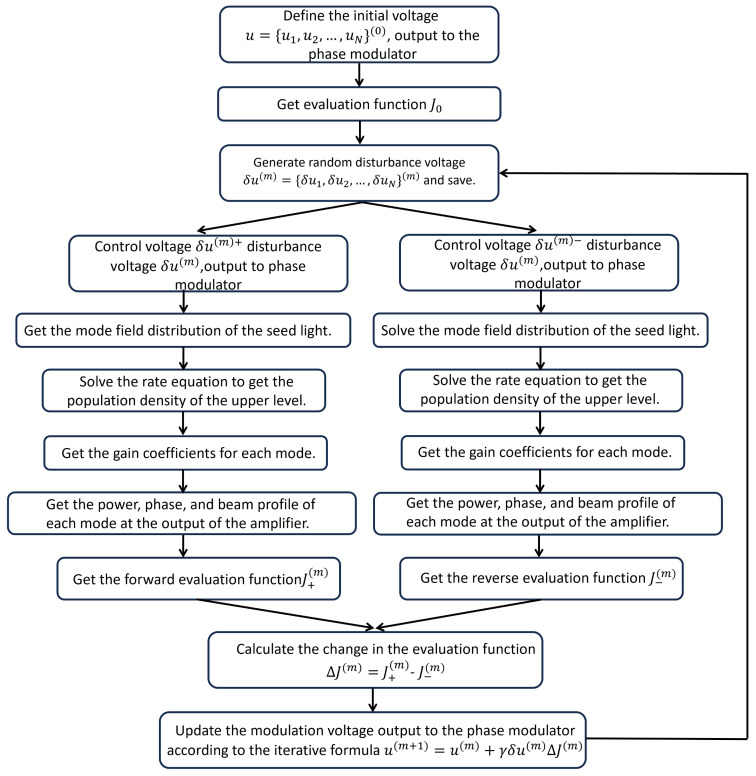
A flowchart delineating the execution of mode control for an adaptive optics system utilizing the SPGD algorithm.

To better understand the simulation process, a flowchart delineating the execution of mode control for an adaptive optics system utilizing the SPGD algorithm is illustrated in Figure 3. The flowchart primarily consists of two sections: the adaptive control section of the photon lantern and the fiber amplifier section. The SPGD algorithm is chosen for adaptive control because it converges faster, has a higher control bandwidth, and offers advantages such as strong scalability and a high power fluctuation tolerance compared to other commonly used optimization algorithms, including multi-dither techniques, genetic algorithms, blind uphill algorithms, and simulated annealing algorithms [20,21]. In the SPGD algorithm, *m* denotes the current iteration number, γ signifies the gain coefficient, and $\delta u^{\left(m\right)}$ represents the magnitude of the random disturbance voltage during the $m^{th}$ iteration. Additionally, ΔJ indicates a critical change in the evaluation function that provides gradient information for iterations, where the evaluation function *J* is chosen as the modal content of the fundamental mode. By generating a random disturbance voltage $\delta u^{\left(m\right)}$ for the input arms of the photonic lantern, the affected mode profile of the seed laser; the population density of the upper level; and the power, phase, and beam profile at the output of the amplifier are determined using Equations (1) through (5). At last, the forward and reverse evaluation functions $J_{\pm }^{m}$ are achieved, and then the modulation voltage output can be updated according to the iterative formula $\mu^{m+1}=\mu^{m}+\gamma \delta \mu^{m}\Delta J^{m}$. In these calculations, the initial phase of each circuit is assigned a random value within the range of 0 to 2π, while the initial amplitude of the input light for each circuit is randomly selected from a range of up to 0.05. The amplitude control frequency is set at 1 kHz, and the algorithm concludes after completing 300 cycles of system iterations.

## 3. Results and Discussions

In this simulation, a 50/400 double-clad YDF (Ytterbium-doped fiber) with a core numerical aperture (NA) of 0.06 is connected to a 6 × 1 photonic lantern. The other physical parameters used in this simulation are listed in Table 1.

### 3.1. The Effect of Mode Competition Within the Gain Fiber on Mode Control Results

**Figure 4:**
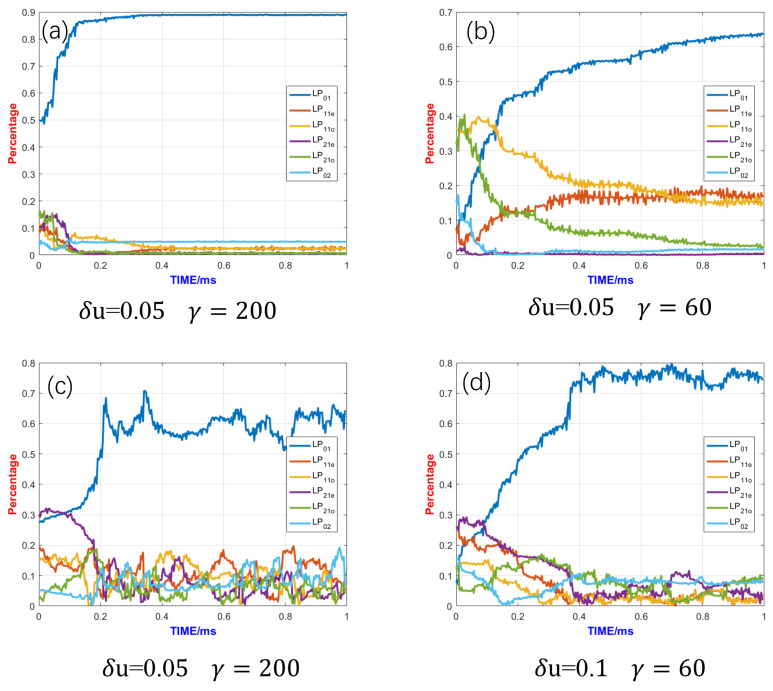

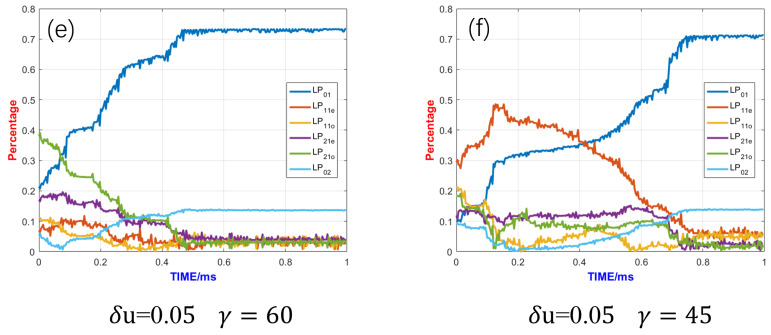
Control effects under different control parameters: (**a**) The control parameters are δu=0.05 and γ=200 without power amplification. (**b**) The control parameters are δu=0.05 and γ=60 without power amplification. (**c**) The control parameters are δu=0.05 and γ=200 with power amplification. (**d**) The control parameters are δu=0.1 and γ=60 with power amplification. (**e**) The control parameters are δu=0.05 and γ=60 with power amplification. (**f**) The control parameters are δu=0.05 and γ=45 with power amplification (Figure(**c**–**f**) were performed at a gain fiber length of 8 m and a pump power of 5 kw).

Since the competition between the fundamental mode and higher-order modes largely determines the beam quality of fiber lasers, performing power scaling during adaptive control can lead to significantly different control outcomes. As shown in Figure 4a, when the adaptive control system is run without power amplification, the fundamental mode content is higher after the algorithm stabilizes, and the beam quality is usually better. Typically, the fundamental mode content after closed-loop control can approach 90%, and the SPGD control converges in a very short time, approximately 0.1 ms, with control parameters γ=200 and δu=0.05. Figure 4b shows the mode control effect without power amplification when γ=200, δu=0.05, the convergence speed of the algorithm is very slow, and convergence is not completed within 1 ms.

However, with the addition of power amplification, the fundamental mode content decreases and the higher-order mode content increases due to mode competition. By examining the fundamental mode content at this point, it can be seen that the fundamental mode content decreases after the control algorithm stabilizes. The optimal control parameters before and after the power scaling of the system are found to be quite different through several trials of the optimal control parameters for different cases. Using the same control parameters, as seen in Figure 4c, mode coupling is generated between the modes, and the fundamental-mode components keep changing over time, indicating poor SPGD control stability.

The comparison of control effects when δu and γ take different values is shown in Figure 4c through Figure 4f. By adjusting these two control parameters, the control stability improves when δu and γ decrease. The perturbation signal and the gain coefficient determine the convergence speed and stability of the algorithm. Generally, the larger $\left|\delta u\right|$ and γ are, the faster the convergence speed and the worse the stability; the smaller $\left|\delta u\right|$ and γ are, the slower the convergence speed and the better the stability. After trying different gain parameters, it is found that when the gain parameters are selected to δu=0.05 and γ=60, this selection takes into account both the convergence speed and stability. As shown in Figure 4e, the fundamental mode content can only approach about 72%, which is 80% of that without power amplification. A lower fundamental mode content results in poorer beam quality. The biggest higher-order mode content is the LP_02_ mode, as the gain of the LP_02_ mode is quite high, making it difficult to suppress effectively. Other methods to suppress the LP_02_ mode should be considered, such as bending the gain fiber. For example, a bending diameter of 18 cm might be a reasonably suitable parameter, as the bending loss of the LP_02_ mode is 0.57 dB/m, which can effectively suppress the LP_02_ mode. If the bending radius of the gain fiber is excessively large, the effective suppression of the LP_02_ mode becomes challenging. Conversely, if the bending radius is too small, it leads to an increase in loss for the fundamental mode. Furthermore, considering practical experimental conditions, a 50/400 double-clad fiber struggles to endure a significantly reduced bending radius. Additionally, as shown in Figure 4d, when δu takes a value of 0.1 and γ is 60, the control effect appears satisfactory. This situation features a faster convergence rate, and the proportion of high-order modes is below 0.1. Nevertheless, the increase in the control parameter δu leads to the instability of the fundamental mode proportion after the algorithm converges, which might result in a temporal jitter of the output beam in experiments. Each of the two control parameters has its own advantages, and the final choice might require a trade-off between the proportion of the fundamental mode seen and control stability.

Furthermore, the convergence time reached 0.45 ms, which is much longer than that without power amplification. A longer convergence time means that faster hardware is needed to ensure the algorithm’s convergence. For future adaptive amplifiers based on photonic lanterns with larger mode fields and higher powers, a 1 MHz control bandwidth may no longer be sufficient, and hardware with a larger control bandwidth will be required.

### 3.2. The Influence of Gain Fiber Length on the Control Effect

The length of the gain fiber simultaneously affects the mode control’s effectiveness and the optical-to-optical efficiency of the photonic lantern adaptive amplifier. A gain fiber length that is too short will result in inadequate pump light absorption and lower pumping efficiency, and a gain length that is too long will result in more susceptible nonlinear effects and control effects. By simulating the effect of different fiber lengths on mode control, the pumping efficiency, fundamental mode content, and m^2^ factor of a control system with different gain fiber lengths are obtained, as shown in Table 2.

As can be seen from Table 2, when the gain fiber length is too short, the pump light cannot be completely absorbed, leading to very low pumping efficiency. When the fiber length reaches 6 m or more, the pumping efficiency becomes essentially stable. When the fiber length exceeds 4 m, the fundamental mode content exceeds 70%. However, in the field of high-power fiber lasers, the focus is on the laser’s brightness. The m^2^ factor is commonly used to evaluate beam quality, and an examination of the m^2^ factor reveals that when the fiber length is 8 m, the m^2^ factor is the smallest, while the fundamental mode content is also relatively high.

**Figure 5:**
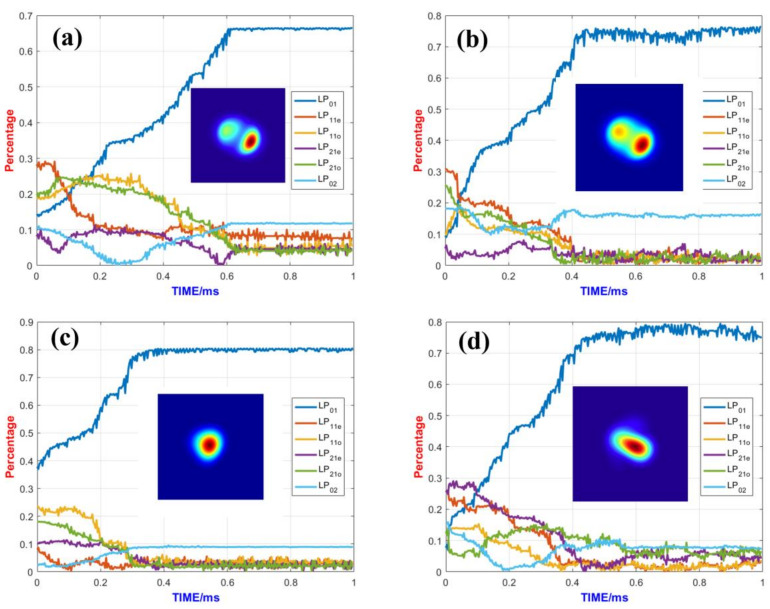
Mode content evolution and output beam for different gain fiber lengths at pump power of 5 kw: (**a**) gain fiber length of 3 m, (**b**) gain fiber length of 5 m, (**c**) gain fiber length of 8 m, and (**d**) gain fiber length of 12 m.

Figure 5 illustrates the evolution of the mode content and the output beam for several typical gain fiber lengths. There is a brief period after the start of system control when multiple modes are coupled to each other, after which the fundamental mode content rises rapidly and remains stable. When the gain fiber is 3 m, the content of the fundamental mode is low (66%) and the output spot has obvious splitting; at this time, the m^2^ factor is 1.6. As the length of the fiber increases, the fundamental mode content increases and the beam quality is optimized. When the fiber length is 8 m, the m^2^ factor reaches 1.22 and the output spot is concentrated in energy and has a good morphology. When the gain fiber is increased to 12 m, the fundamental mode content does not significantly improve and the output spot morphology is distorted, with the m^2^ factor degrading to approximately 1.4. Therefore, the optimal gain fiber length for the system is 8 m.

### 3.3. The Influence of Pumping Methods on the Control Effect

**Figure 6:**
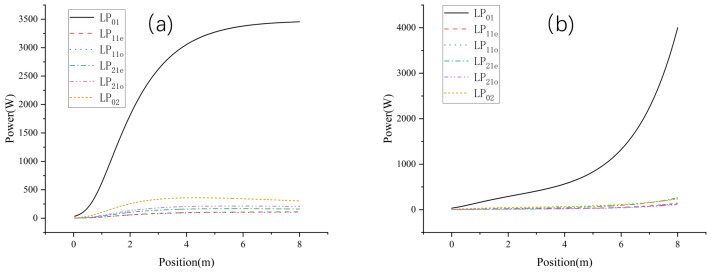
Distribution of optical power in the fiber using different pumping methods: (**a**) forward pumping and (**b**) backward pumping.

The impact of different pumping methods on the high-power mode control system is primarily reflected in the system’s pumping efficiency and the impact of nonlinear effects. Figure 6 illustrates the optical power distribution within the gain fiber under different pumping methods, and, ultimately, a higher output power is obtained with backward pumping. Backward pumping achieves higher pumping efficiency compared to forward pumping. From the comparison in Figure 6, we can see that for the forward-pumped photonic lantern adaptive amplifier, the $LP_{02}$ mode reaches its maximum stable value within a very short fiber length, whereas for the backward-pumped amplifier, the $LP_{02}$ mode does not reach its maximum stable value. The powers of the $LP_{02}$ mode for forward and backward pumping are 303 W and 180 W, respectively.

**Figure 7:**
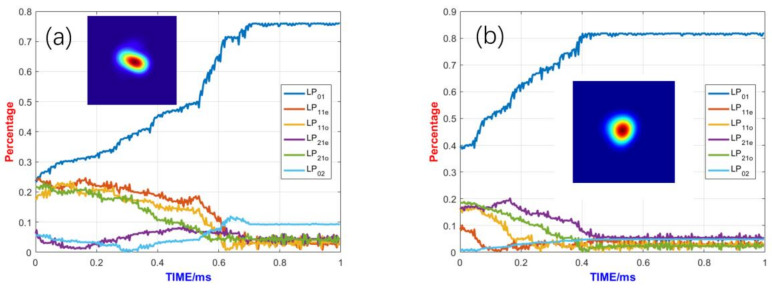
Comparison of control effects of different pumping methods: (**a**) forward pumping and (**b**) backward pumping.

In addition, Figure 7 shows the control effect of the fundamental mode under different pumping methods. For the forward pumping method, the fundamental mode content approaches 75% and the output spot morphology is distorted. However, for the backward pumping method, the fundamental mode content approaches 81% and the output beam profile is closer to the fundamental mode beam profile. In addition, the convergence time of the backward pumping method is much shorter than that of forward pumping method. Therefore, backward pumping is a superior pumping method for achieving better mode control results.

### 3.4. The Influence of Gain Fiber Pumping Power on the Control Effect

**Figure 8:**
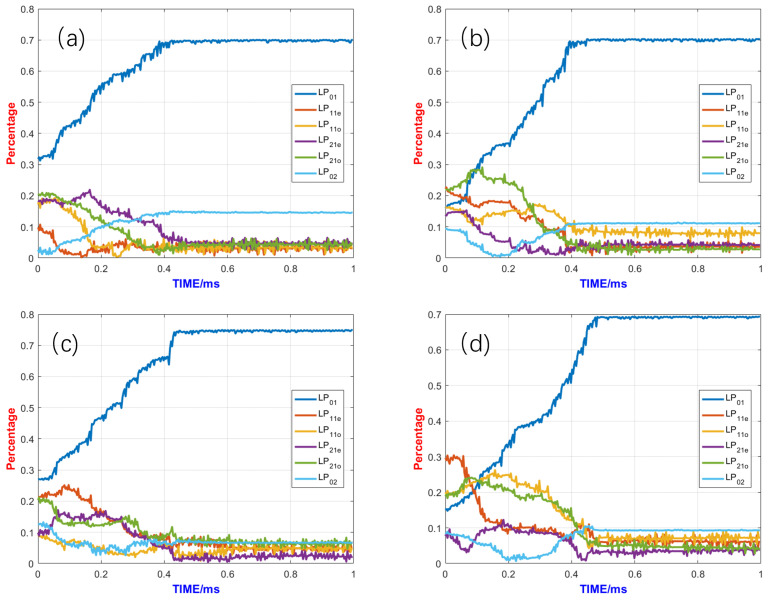
Comparison of control effects under different pump powers: (**a**) the pump power is 50 W, (**b**) the pump power is 1000 W, (**c**) the pump power is 3000 W, and (**d**) the pump power is 5000 W.

To verify the power amplification potential of the system, the mode control effects seen under different pumping powers were compared in this study. The control effects under different pumping powers are shown in Figure 8. From the comparison of the mode control effects under different pumping powers, it can be seen that the adaptive control system can achieve good control effects under pumping powers ranging from 50 W to 5 kW. After the control algorithm starts, the content of the fundamental mode rapidly increases, while other higher-order modes are suppressed. After stabilizing, the content of the fundamental mode exceeded 70% in all cases. This demonstrates that the system has significant power amplification potential and can stabilize mode control under different pumping powers.

## 4. Conclusions

An adaptive control method for fiber modes based on a photonic lantern has been proven to be effective in achieving the adaptive control of fiber modes both theoretically and experimentally. Experimentally, a near-fundamental-mode output has been achieved for kilowatt-level large-mode-field fiber lasers [18]. Theoretically, simulations of mode control in photonic lanterns have primarily focused on mode control within the photonic lantern devices themselves, without considering the impact of fiber amplifiers. In this paper, we combine the gains and losses of individual modes within the fiber amplifier and introduce a mode content parameter at the amplifier’s output as an evaluation function to simulate the mode control effects of a photonic lantern amplifier. The competition between different modes within the gain fiber can lead to varying control effects compared to the simulation of mode control in photonic lantern devices. Using the same control parameters, mode coupling is generated between the modes and the fundamental mode components keep changing over time, indicating poor SPGD control stability. By selecting appropriate control parameters, the fundamental mode content can only approach about 72%, which is 80% of that seen without power amplification. A lower fundamental mode content results in poorer beam quality. The biggest higher-order mode content is the LP_02_ mode, as the gain of the LP_02_ mode is quite high, making it difficult to suppress effectively. Therefore, other methods should be considered to suppress the LP_02_ mode. Furthermore, the convergence time is much longer than that without power amplification. A longer convergence time means that faster hardware is needed to ensure the algorithm’s convergence. In summary, the mode competition within the gain fiber can degrade the control effect of the fundamental mode and cause the control to take a longer time to converge.

Additionally, we studied how the parameters of the fiber laser affect the control effectiveness of the adaptive fiber amplifier based on a photonic lantern. The length of the gain fiber simultaneously affects the mode control’s effectiveness and the optical-to-optical efficiency of the photonic lantern adaptive amplifier. A gain fiber length that is too short results in inadequate pump light absorption and lower pumping efficiency, while a gain fiber length that is too long makes the system more susceptible to nonlinear effects and diminishes the control effect. Through optimization and comparison, it can be concluded that the optimal gain fiber length for the system is 8 m. In addition, we also investigated the influence of pumping methods on the control effect. The backward pumping method achieves higher pumping efficiency and better control results. The influence of the gain fiber pumping power on the control effect was also studied in this work. From the comparison of the mode control effects under different pumping powers, it can be seen that the adaptive control system can achieve good control effects under pumping powers ranging from 50 W to 5 kW. This demonstrates that the system has significant power amplification potential and can stabilize mode control under different pumping powers.

In conclusion, our research demonstrates that an adaptive fiber amplifier based on a photonic lantern can achieve a stable, high-power, large-mode-field, near-fundamental-mode output from the gain fiber. Although mode competition within the gain fiber can degrade the control effect of the fundamental mode and cause the control to take a longer time to converge, these aspects should be further studied to improve the control’s effectiveness. As research progresses, these simulation models will continue to evolve, not only providing greater guidance for high-power mode control experiments but also deepening our understanding of physical processes and driving innovation in science and technology.

## Figures and Tables

**Table 1:** Physical parameters exploited in this simulation.

Parameter	Value
Core radius, $r_{1}$	25 μm
Inner cladding radius, $r_{2}$	200 μm
Outer cladding radius, $r_{3}$	250 μm
Doping concentration of Yb ion	$2\times 10^{25}$/m^3^
Pumping power	5000 W
Signal light wavelength, $\lambda_{s}$	1064 μm
Pumping wavelength, $\lambda_{p}$	976 μm
Refractive index of fiber, $n_{co}$	1.4589
Refractive index of inner cladding, $n_{cl}$	1.4577
Spontaneous lifetime, τ	900 μs
Pump absorption cross-section, $\sigma_{ap}$	$2.4\times 10^{-24}$ m^2^
Pump emission cross-section, $\sigma_{ep}$	$2.4\times 10^{-24}$ m^2^
Signal absorption cross-section, $\sigma_{as}$	$3.6\times 10^{-27}$ m^2^
Signal emission cross-section, $\sigma_{es}$	$4.3\times 10^{-25}$ m^2^

**Table 2:** Effect of gain fiber length on pumping efficiency, fundamental mode content, and m^2^ factor.

Gain Fiber Length	Pumping Efficiency	Fundamental Ratio	m^2^ Factor
3 m	53.5%	66%	1.6
4 m	58.3%	71.8%	1.44
5 m	64.8%	73%	1.3
6 m	70.6%	75%	1.33
7 m	72.4%	76%	1.33
8 m	72.4%	79%	1.22
9 m	73.1%	74%	1.34
10 m	71%	71%	1.42
11 m	70.6%	71%	1.43
12 m	70.2%	72%	1.4

## Data Availability

The original contributions presented in this study are included in the article. Further inquiries can be directed to the corresponding author(s).
